# Colloidal Particles in Tuna Head Soup: Chemical Localization, Structural Change, and Antioxidant Property

**DOI:** 10.3389/fnut.2021.638390

**Published:** 2021-03-29

**Authors:** Chenchen Ma, Pingping Liu, Ningping Tao, Xichang Wang, Shanggui Deng

**Affiliations:** ^1^National R&D Branch Center for Freshwater Aquatic Products Processing Technology (Shanghai), Integrated Scientific Research Base on Comprehensive Utilization Technology for By-Products of Aquatic Product Processing, Ministry of Agriculture and Rural Affairs of the People's Republic of China, Shanghai Engineering Research Center of Aquatic-Product Processing and Preservation, College of Food Science & Technology, Shanghai Ocean University, Shanghai, China; ^2^College of Food and Pharmacy, Zhejiang Ocean University, Zhoushan, China

**Keywords:** antioxidant property, core-shell structure, human umbilical vein endothelial cells, particle size, tuna colloidal particles

## Abstract

In this work, chemical localization, structural changes, and antioxidant properties of tuna colloidal particles (TCPs) in boiling tuna head soup were examined. The results demonstrated that TCPs might be core–shell colloidal spherical structures. The hydrophobic core consisted of triglycerides and chloride ions. The hydrophilic shell layer consisted of chloride ions, sodium ions, phospholipids, protein, and glycosyl molecules. Coalescence of TCPs occurred during the boiling process, and water may enter the hydrophobic core of TCPs after the boiling time of 60 min. TCPs had excellent antioxidant properties against H_2_O_2_-induced human umbilical vein endothelial cell injury. It might be resulted from that TCPs could decrease cell apoptosis proportion and downregulate mRNA levels of endoplasmic reticulum-bounded chaperone protein glucose-related protein (GRP78), C/EBP homologous protein (CHOP), and activating transcription factor-4 (ATF4). This work can provide useful basic information to understand the colloidal system in foods, especially in soup. In addition, it may also promote the potential high-value-added utilization of aquatic by-products.

## Introduction

Tuna is a diverse family of marine fish and widely distributed in the tropical and subtropical waters of the major oceans such as the North Atlantic and Indian Ocean (1). Among the tuna family, bigeye tuna (*Thunnus obesus*) is one of the major species on the global tuna market (2). Tuna is rich in protein, vitamins, minerals, and omega-3 unsaturated fatty acids such as docosahexaenoic acid (DHA) and eicosapentaenoic acid (EPA). Omega-3 unsaturated fatty acids can lower blood lipids, regulate biochemical and physiological reactions, activate brain cells, enhance memory, promote brain development in infants, increase anti-inflammatory abilities, reduce the risk of coronary diseases, and prevent certain cancers (3, 4).

Tuna is commonly processed into raw sashimi and steak. The processing generates many by-products including head, viscera, gills, dark flesh, skin, and bone. These by-products compose almost 50–70% weight of the original tuna. High-value-added utilization of these by-products has attracted much attention in the field of food and aquatic products. Fish by-products have been explored and applied to produce fish oil, fishmeal, fertilizer, fish silage, profitable bioactive compounds (i.e., bioactive peptides, oligosaccharides, fatty acids, enzymes, and water-soluble minerals) (5). Tuna by-products have also been explored and applied to produce fish oil (6), fishmeal (7), antioxidative peptides (8), collagen (9), sulfated polysaccharides (10), etc. These works significantly promoted the research and development of high-value-added utilization of tuna by-products. However, more researches are needed to deeply and widely explore the high-value-added utilization of tuna by-products.

Soup is one of the most popular diets in the world because of its significant advantages such as convenient processing, flavorful taste, and easy absorption to people (11). Soup can dissolve and retain many food nutrients from the food materials. It is especially suitable for the elderly, children, infants, and the infirm. Therefore, tuna head soup may be a simple way for high-value-added utilization of tuna head. Migration of nutritional components (e.g., lipid, protein, sugar, and other components) in tuna head soup and the effect of processing methods on them are necessary to be illustrated, which will be helpful to understanding the nutritional mechanism of tuna head soup for human beings. Our previous works found that micro/nanosized colloidal particles were formed in Atlantic salmon, bighead carp, and tuna head soups (12–14). After a series of chemical and physical reactions in the boiling process of tuna head soup, the chemical components included not only the original components migrated from the tuna head but also some newly formed components. These components could self-assemble to form micro-nano-colloidal particles during the boiling process. Further works are needed to deeply analyze the chemical localization and structural stability of colloidal particles during the boiling process and their potential antioxidant properties for human health.

Human umbilical vein endothelial cell (HUVEC) damage has been recognized to be associated with cardiovascular diseases. The simulation of H_2_O_2_, one of the most widely studied reactive oxygen species, or its intracellular production is responsible for the activation or deregulation of various signaling pathways, and participate in the initiation and development of cardiovascular diseases such as hypertension and atherosclerosis (15, 16). Therefore, H_2_O_2_-induced oxidative injury of HUVECs is a good cell model to study the antioxidant properties of some newly found substances (17–19).

Here, we examined the chemical localization and structural changes of tuna colloidal particles (TCPs) in boiling tuna head soup by laser scanning confocal microscopy, laser light scattering technique, and transmission electron microscopy. Further, we analyzed the antioxidant properties of TCPs against H_2_O_2_-induced oxidative injury of HUVECs using CCK-8 assay, real-time PCR, and flow cytometry assay. This work provided useful basic information to understanding the colloidal system in foods, especially in soup.

## Materials and Methods

### Preparation of Tuna Head Soup

Tuna head soups were prepared according to our previous work with minor revision (12). Frozen half bigeye tuna heads (length: 27 ± 2 cm; width: 26 ± 2 cm; weight: 1.5 ± 0.3 kg; Dalian Xiangxiang Food Co., Ltd., Liaoning, China) were defrosted using a running-water thawing method for 1 h. Then, the tuna heads were washed with ultrapure water and cut into small pieces (5 × 3 × 2 cm). The tuna head pieces were drained to remove the ultrapure water. Subsequently, 400-g tuna head pieces were fried in 20 g cooked soybean oil at 120°C for 30 s on a home-use induction cooker (2,400 W). Then, 3,200 mL ultrapure water was immediately added to the fried tuna head pieces. After 10.5 min, the water was boiled. The tuna head piece–water mixtures were kept boiled at the temperature of 90°C for 150 min. The control sample was prepared using the same procedure without the addition of tuna head pieces. At the designed time points, 50 mL of soup was taken out and filtered for below research. Six parallel experiments were done for each sample.

### Laser Scanning Confocal Microscopic Observation of TCPs

The LSCM observation was performed according to our and others' previous works with minor revision (20–24). Nile Red is a selective stain for lipids. N-[Ethoxycarbonylmethyl]-6-methoxy-quinolinium bromide (MQAE) is a fluorescent indicator for intracellular chloride ions (Cl^−^). CoroNa™ Green is a sodium ion indicator that exhibits an increase in green fluorescence emission intensity upon binding sodium ions (Na^+^), with little shift in wavelength. Therefore, Nile Red, MQAE, and CoroNa™ Green could specifically stain TG, chloride ions, and sodium ions, respectively. Rd-DOPE is a polar head group-labeled phospholipid probe. Nile Blue (Shanghai Macklin Biochemical Co., Ltd., China) might be in neutral form and could only bind to protein. Wheat germ agglutinin Alexa Fluor 488 (WGA488) can bind to sialic acid and N-acetylglucosaminyl residues to stain glycosyl molecules. Therefore, Rd-DOPE, Nile Blue, and WGA488 could specifically stain phospholipids (PL), proteins, and glycosyl molecules (GM), respectively.

In this work, freshly prepared tuna colloidal particle solutions (1 mL) were mixed with 100 μL of Nile Red (Sangon Biotech Shanghai Co., Ltd., Shanghai, China), 40 μL of MQAE (MedChemExpress, USA), and 40 μL of CoroNa™ Green (Thermo Fisher Scientific, USA) to stain the triglycerides, chloride ions, and sodium ions, respectively. Freshly prepared tuna colloidal particle solutions (1 mL) were mixed with 20 μL of Rd-DOPE (Avanti Polar Lipids, Inc. Alabaster, AL, USA), 10 μL of Nile Blue (Sangon Biotech Shanghai Co., Ltd., Shanghai, China), and 10 μL of WGA488 (Biotium, San Francisco, CA, USA) to stain the phospholipids, proteins, and glycosyl molecules, respectively. These samples were incubated in the dark for 30 min. Then, 10 μL of the stained samples was added onto glass microslides and square cover glasses were placed onto the samples. Finally, these samples were observed using a LSCM microscope (Zeiss LSM 710, Carl Zeiss, Jena, Germany) with a 63× (NA 1.4) oil immersion objective. LSCM observation was performed using an argon laser (excitation wavelength of 488 nm, emission wavelength of 500–535 nm), a He–Ne laser (excitation wavelength of 543 nm, emission wavelength of 565–615 nm), and a diode laser (excitation wavelength of 633 nm, emission wavelength of >650 nm).

### Size Distribution of TCPs

The size distribution of TCPs in the soup was measured by laser light scattering technique using a Mastersizer 2000 instrument (Malvern, UK). The size range was set from 0.020 to 2,000 μm. The refractive indices of 1.520 and 1.330 were used for the particle and dispersant, respectively (25, 26). Standard parameters were calculated by the software (27): the surface weighted mean [$d_{32}=\sum v_{i}/\sum \left(\frac{v_{i}}{d_{i}}\right)$, where *v*_*i*_ is the volume of particles in a size class of average diameter of *d*_*i*_]; the volumic weighted mean [*d*_32_ = ∑(*v*_*i*_ × *d*_*i*_)/∑*v*_*i*_]; the specific surface area ($=6\times \rho^{-1}\times {d_{32}}^{-1}$, where ρ is the solution density); and the span distribution [*span* = (*d*_0.9_ − *d*_0.1_)/*d*_0.5_, where *d*_0.1_, *d*_0.5_, and *d*_0.9_ are the diameters below which lie 10, 50, and 90%, respectively, of the particle volumes].

### Transmission Electron Microscopic Observation of TCPs

Freshly prepared tuna colloidal particle solutions were centrifuged at 3,821× g for 2 min at 4°C in an H1850R Table Top High-Speed Centrifuge (Hunan Xiangyi Laboratory Instrument Development Co. Ltd., Changsha City, Hunan Province, China). The supernatants were loaded on a 300-mesh copper grid and then were negatively stained by a droplet of 1% phosphotungstic acid. After drying the grid at room temperature for 5 min, the samples were observed by a transmission electron microscope (Tecnai G2 Spirit Bio Twin, FEI company, USA) at 120 kV acceleration voltage (28). The numbers of the black spots per square micron core part (oil phase) in the TEM images were calculated by analyzing three different TEM images from different samples.

### Cell Culture and Treatments

HUVECs were acquired from the Cell Resource Center of Shanghai Institutes for Biological Sciences, Chinese Academy of Sciences (Shanghai, China). The cells were cultured in Dulbecco's Modified Eagle Medium (DMEM, Gibco, USA) supplemented with 10% fetal bovine serum (FBS, Gibco, USA) and 1% penicillin–streptomycin liquid (Gibco, USA) at 37°C in a humidified atmosphere with 5% CO_2_. The cells were digested with 0.25% trypsin, rinsed with PBS, centrifuged, and harvested in a centrifuge (150 × g at room temperature for 5 min) and resuspended in DMEM. The cells were seeded in 6-well plates at a density of about 1 × 10^6^ cells per well. Then, the cells were cultured at 37°C with 5% CO_2_ for 12 h. The cell wells were randomly divided into five groups (six wells per group) and incubated by fresh media with different substances: control group (control sample), H_2_O_2_ group (50 μM H_2_O_2_), low-dose group (1 μg/mL TCP + 50 μM H_2_O_2_), middle-dose group (10 μg/mL TCP + 50 μM H_2_O_2_), and high-dose group (100 μg/mL TCP + 50 μM H_2_O_2_). TCPs in tuna head soup at the boiling time of 150 min were applied in this assay. In this process, after 24 h incubation with TCP, the cells were incubated with H_2_O_2_ for 2 h. After H_2_O_2_ incubation, the cells were analyzed by cell viability assay, real-time PCR assay, and flow cytometry assay.

### Cell Viability Assay

The cell viability of HUVECs was examined by CCK-8 assay according to previous established methods (29, 30). Briefly, the culture media were removed and fresh media containing 10% CCK-8 solution were added to each well and incubated for 4 h at 37°C. After that, 100 μL of supernatant was transferred into a 96-well plate. The absorbances were measured at 450 nm using a microplate reader (Multiskan MK3, Thermo Labsystems, Finland). Six parallel experiments were performed for each group.

### Real-Time PCR

Total RNAs were isolated using a High Pure RNA Isolation Kit (Roche, Germany) according to the manufacturer's protocol. Then, cDNAs were synthesized using a RevertAid First Strand cDNA Synthesis Kit (Fermentas, USA). The relative mRNA levels of the genes in HUVECs were measured using a Faststart Universal SYBR Green Master (Roche, Germany) on a LightCycler 480 instrument (Roche, Germany). All quantifications were performed with β-actin as an internal standard. The relative amount of mRNA was calculated using the relative quantification (ΔΔCT) method (31). The relative amount of each mRNA was normalized to that in control samples. Quantitative RT-PCR assay was performed using specific primers against the following target genes (32): GRP78 (forward, 5′-CACGTCCAACCCGGAGAA-3′; reverse, 5′-TTCCAAGTGCGTCCGATGA-3′); CHOP (forward, 5′-ACCAAGGGAGAACCAGGAAACG-3′; reverse, 5′-TCACCATTCGGTCAATCAGAGC-3′); ATF4 (forward, 5′-AAACCTCATGGGTTCTCCAG-3′; reverse, 5′-GGCATGGTTTCCAGGTCATC-3′).

### Flow Cytometry Assay

For apoptosis analysis, quantification of the cells was examined using the Annexin-V-FITC/Propidium Iodide (PI) Apoptosis Detection Kit (BD Company, Franklin Lakes, New Jersey, USA) according to the manufacturer's protocols (33). Briefly, after the treatment, the culture media were removed and the cells were digested with fresh media with 0.25% trypsin, rinsed with cold PBS twice, centrifuged and harvested in a centrifuge (150× g at room temperature for 5 min), and resuspended in fresh media with Annexin-V-FITC and PI at room temperature. After incubation for 15 min in the dark, these samples were analyzed using a flow cytometer (BD, USA).

### Statistical Analysis

The data are expressed as the means ± standard deviations (SD). The statistical differences were calculated using the Student's *t*-test. The data were considered statistically significant when *P* < 0.05.

## Results

### Chemical Localization of TCPs in Tuna Head Soup

**Figure 1:**
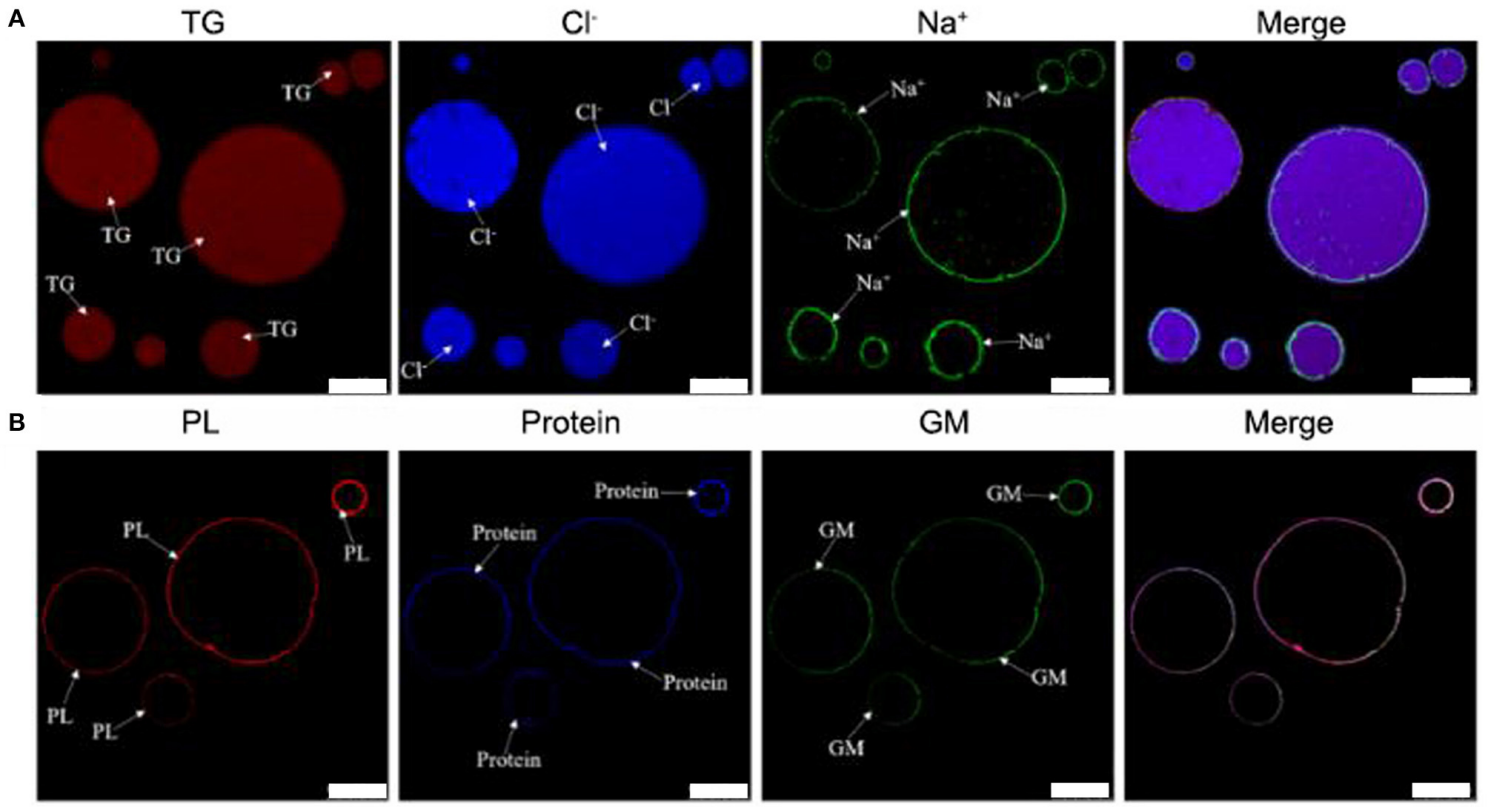
LSCM images of TCPs in tuna head soup at 150 min boiling time. **(A)** Nile Red, MQAE, and CoroNa™ Green were used to stain the triglycerides (TG), chloride ions (Cl^−^), and sodium ions (Na^+^), respectively. **(B)** Rd-DOPE, Nile Blue, and WGA488 were used to stain the phospholipids (PL), proteins, and glycosyl molecules (GM), respectively. White scale bars indicate 10 μm.

Previous work suggested TCPs were micro/nano-sized colloidal structures (12). However, the location of chemical compositions was not systematically analyzed by co-localization technique of LSCM. In addition, the location of sodium and chloride ions was also not analyzed by LSCM. In order to deeply analyze the chemical localization of TCPs in tuna head soup, the co-localization technique of LSCM was applied in this work. As shown in Figure 1, the chemical localization of TCPs in tuna head soup at the boiling time of 150 min was analyzed by LSCM. According to the results, triglyceride ions were present in the inner core part of colloidal particles. Sodium ions, phospholipids, proteins, and glycosyl molecules were mainly present in the periphery part of colloidal particles. Chloride ions were present both in the inner core part and in the periphery part of colloidal particles. Therefore, the TCPs might be core–shell colloidal spherical structures, just like droplet structures in emulsions (20). The core part might be hydrophobic and consisted of TG and chloride ions. The shell layer might be hydrophilic and consisted of chloride ions, sodium ions, phospholipids, protein, and glycosyl molecules.

### Size Distribution and Structure Changes of TCPs in Tuna Head Soup During the Boiling Process

**Figure 2:**
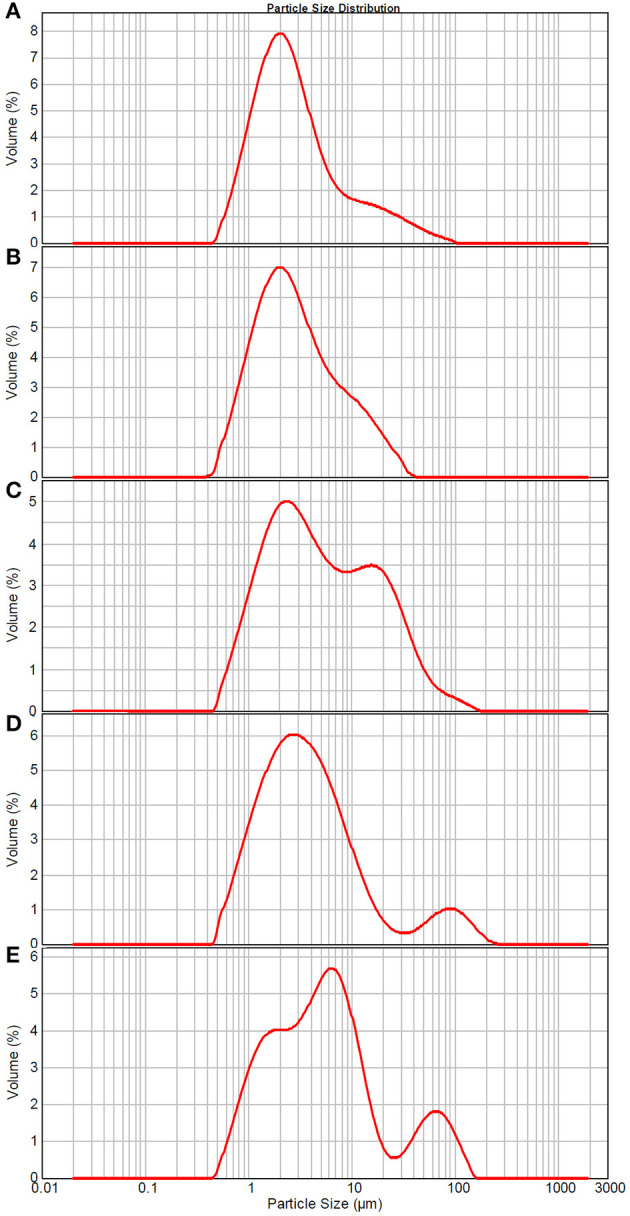
Size distribution of TCPs in tuna head soup at different boiling times. **(A)** 5 min; **(B)** 15 min; **(C)** 30 min; **(D)** 60 min; and **(E)** 150 min.

The particle size distribution and structure changes of TCPs in tuna head soup during the boiling process were measured by the Malvern size analyzer and TEM, respectively. As shown in Figure 2, TCPs with diameters of 0.5–100 μm appeared in tuna head soup at the boiling time of 5 min. With the increase in boiling time, particle size increased and three peaks appeared in the size distribution image at the boiling time of 150 min (Figure 2).

**Figure 3:**
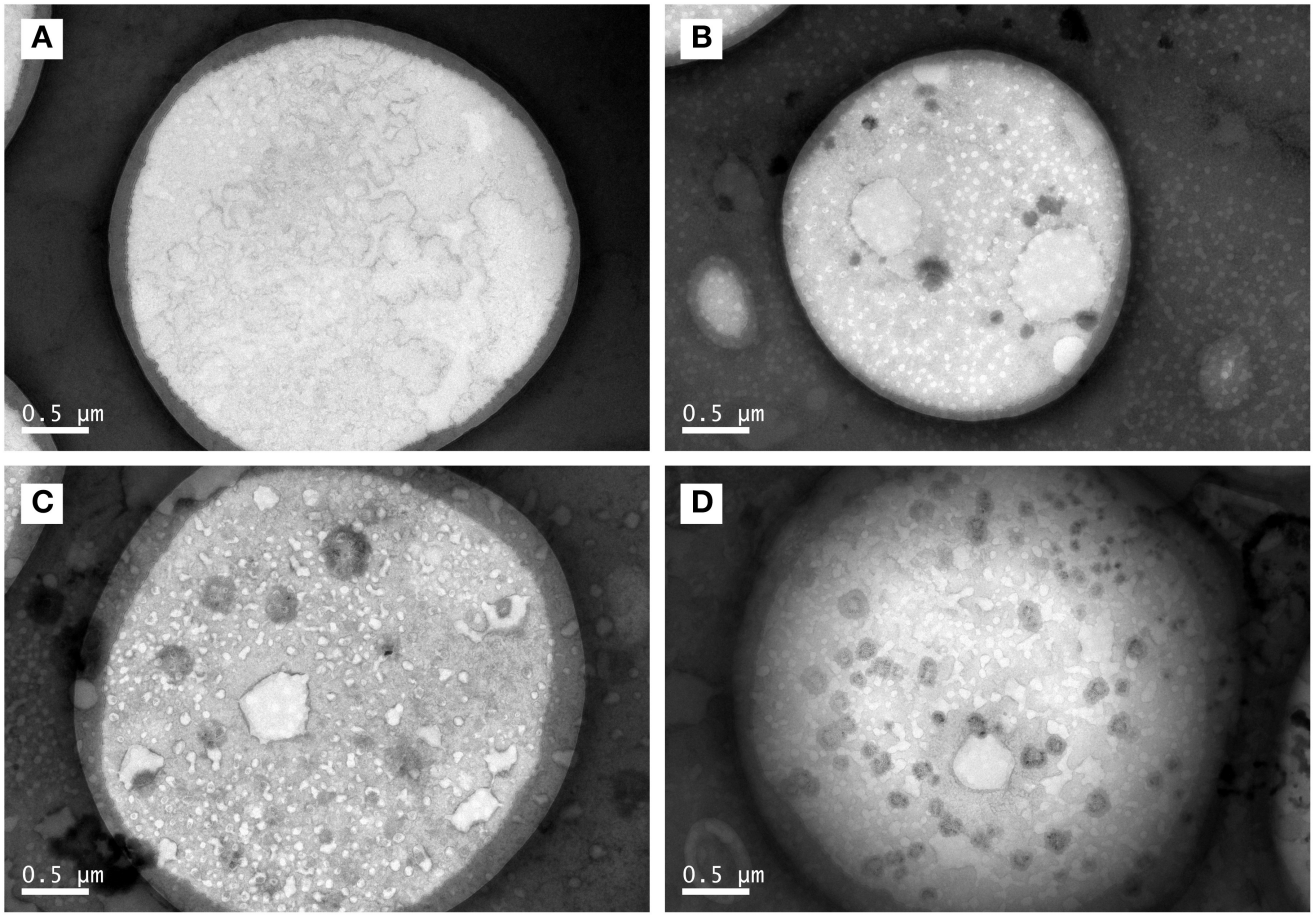
TEM images of TCPs in tuna head soup at different boiling times. **(A)** 30 min; **(B)** 60 min; **(C)** 90 min; and **(D)** 150 min. Scale bars indicate 0.5 μm.

As shown in Figure 3, the core–shell structures that were suggested by LSCM results (Figure 1) were confirmed by representative TEM images. Moreover, some black spots appeared in the core part of TCPs at the boiling time of 60 min and in the shell layer part of TCPs at the boiling time of 90 min. The numbers of the black spots per square micron core part (oil phase) in the TEM images were 0.04 ± 0.06, 1.60 ± 0.67, 2.26 ± 0.41, and 4.13 ± 0.84 for 30, 60, 90, and 150 min, respectively. Therefore, the amounts of black spots increased with the increase of boiling time.

### Antioxidant Properties of TCPs Against H_2_O_2_-Induced HUVEC Injury

**Figure 4:**
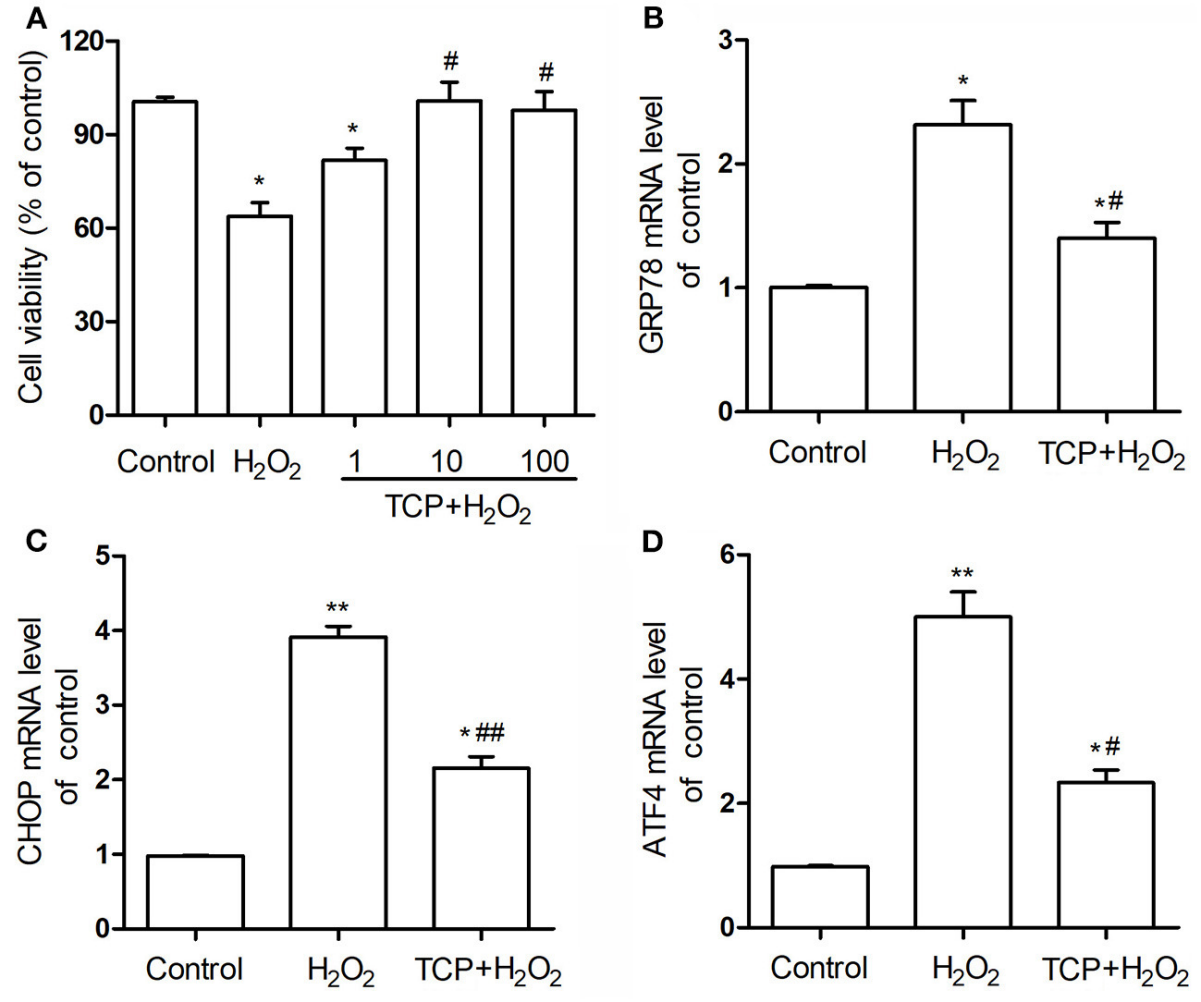
Cell viability **(A)** of TCP-protected HUVECs with different TCP concentrations (1, 10, and 100 μg/mL) and mRNA expression levels of GRP78 **(B)**, CHOP **(C)**, and ATF4 **(D)** of 10 μg/mL TCP-protected HUVECs against H_2_O_2_-induced injury. The data are expressed as the means ± SD. Statistically significant differences among different groups: *P < 0.05, **P < 0.01, compared to the control group; ^#^*P* < 0.05, ^##^*P* < 0.001, compared to the H_2_O_2_ group. TCPs in tuna head soup at the boiling time of 150 min were applied in this assay.

In order to analyze the antioxidant properties of TCPs against H_2_O_2_-induced HUVEC injury, CCK-8 cell viability assay was examined. As shown in Figure 4A, the cell viability assay showed that H_2_O_2_ treatment could induce significant HUVEC injury compared with the control group, which confirmed that H_2_O_2_-induced oxidative injury of HUVECs is a good cell model to study the antioxidant properties of some newly founded substances (17–19). TCP pretreatments with different concentrations could significantly decrease H_2_O_2_-induced oxidative injury. Moreover, middle-dose (10 μg/mL TCP + 50 μM H_2_O_2_) and high-dose (100 μg/mL TCP + 50 μM H_2_O_2_) groups showed no obvious differences to the control group, which suggested that middle-dose and high-dose TCP pretreatments could completely inhibit H_2_O_2_-induced oxidative injury. It demonstrated that TCPs had significant antioxidant properties against H_2_O_2_-induced HUVEC injury.

In this work, mRNA expression levels of GRP78, CHOP, and ATF4 of 10 μg/mL TCP-protected HUVECs against H_2_O_2_-induced injury were examined by real-time PCR assay (Figures 4B–D). H_2_O_2_ treatment could significantly upregulate these three mRNA levels compared with the control group, which confirmed that H_2_O_2_-induced oxidative injury of HUVECs was involved in the enhancement of endoplasmic reticulum stress. TCP pretreatments could significantly downregulate these three mRNA levels. However, TCP pretreatments could not decrease the mRNA levels to the values of the control group.

**Figure 5:**
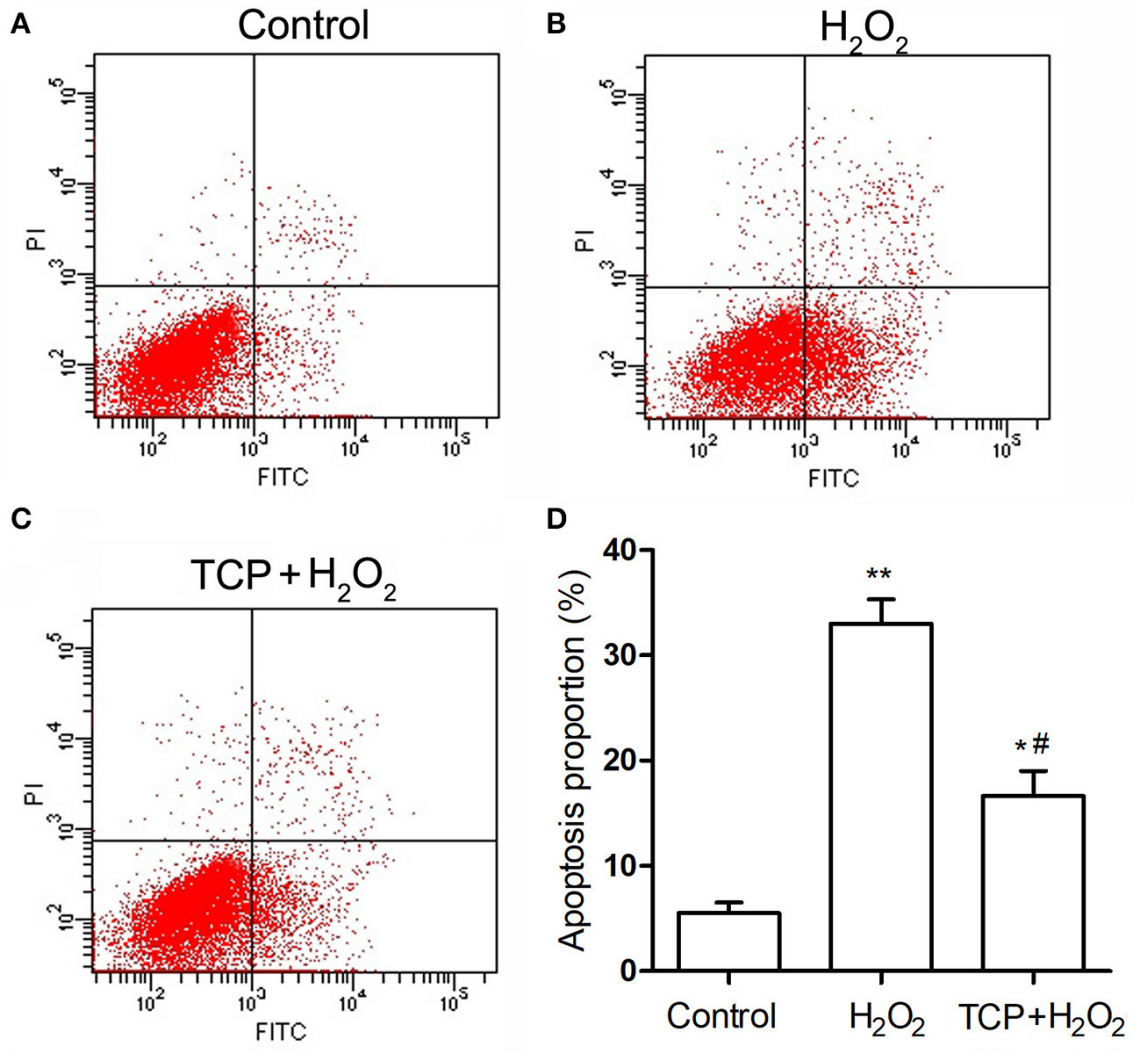
Apoptosis of TCP-protected HUVECs against H_2_O_2_-induced injury. **(A)** Control group. **(B)** H_2_O_2_ group. **(C)** 10 μg/mL TCP + 50 μM H_2_O_2_ group. **(D)** Apoptosis proportion of TCP-protected HUVECs against H_2_O_2_-induced injury. The data are expressed as the means ± SD. Statistically significant differences among different groups: *P < 0.05, **P < 0.01, compared to the control group; ^#^*P* < 0.05, compared to the H_2_O_2_ group. TCPs in tuna head soup at the boiling time of 150 min were applied in this assay.

Finally, in order to analyze cell apoptosis, TCP-protected HUVECs against H_2_O_2_-induced injury were examined by flow cytometry assay (Figure 5). H_2_O_2_ treatment could significantly increase apoptosis proportion (33.1 ± 2.4%) compared with the control group (5.5 ± 1.0%), which confirmed that H_2_O_2_ treatment could significantly induce cell apoptosis of HUVECs. TCP pretreatments could significantly decrease apoptosis proportion (16.8 ± 2.4%) compared with the H_2_O_2_ group. However, pretreatment of TCPs could not decrease the apoptosis proportion to the value of the control group, which is similar to mRNA results (Figure 4).

## Discussion

TCPs were suggested to be micro/nano-sized colloidal structures in previous work (12). However, the chemical localization of TCPs, structural change of TCPs during the boiling process, and antioxidant properties of TCPs have not been studied. In this work, we used LSCM to observe the chemical localization of TCPs (Figure 1). Then, we used the Malvern size analyzer (Figure 2) and TEM (Figure 3) to analyze the particle size distribution and structure changes of TCPs in the tuna head soup during the boiling process. Finally, we used CCK-8 cell viability assay (Figure 4A), real-time PCR assay (Figures 4B–D), and flow cytometry assay (Figure 5).

The LSCM results (Figure 1) suggested that the TCPs might be core–shell colloidal spherical structures, just like droplet structures in emulsions (20). It further confirmed a previous conclusion that TCPs were the micro/nano-sized colloidal structures in previous work (12). Moreover, the core part of TCPs might be hydrophobic and consisted of TG and chloride ions. The shell layer of TCPs might be hydrophilic and consisted of chloride ions, sodium ions, phospholipids, protein, and glycosyl molecules.

Malvern size analyzer results (Figure 2) showed that TCP particle size increased with the increase of boiling time. Moreover, three peaks appeared in the size distribution image at the boiling time of 150 min. The appearance of three peaks might be coalescence of colloidal particles, which is a common phenomenon of colloidal systems (34–36). Coalescence occurs when two or more colloidal particles come into close proximity and fuse together to form a large colloidal particle. It tends to occur when the attractive forces between two particles are larger than the repulsive forces, and then the interfacial layers rupture and fuse together.

Further, the core–shell structures were confirmed by representative TEM images (Figure 3). Moreover, the numbers of the black spots per square micro core part (core phase) in the TEM images increased with the increase of boiling time. LSCM images (Figure 1) of TCPs at the boiling time of 150 min showed that no hydrophilic substances (e.g., proteins, phospholipids, glycosyl molecules, sodium ions, and chloride ions) which were distributed into the core part of TCPs. Therefore, these black spots might not be these hydrophilic substances. Generally, in the process of boiling, the water is fully agitated and actively enter TCPs or passively be involved into the particle coalescence. Therefore, we could reasonably assume that the black spots might be water. It should be noted that this phenomenon only occurred at and after the boiling time of 60 min. Further studies are needed to illustrate the underlying mechanism.

CCK-8 cell viability assay (Figure 4A) showed that TCP pretreatments with different concentrations could significantly decrease H_2_O_2_-induced oxidative injury. As the boiling time increases, the antioxidant performances of colloidal particles are expected to decrease, as they are oxidized over time. Therefore, we only used TCPs in tuna head soup at the boiling time of 150 min in this assay. Therefore, we may predict that TCPs in tuna head soup at the boiling times of 30–150 min had significant antioxidant properties against H_2_O_2_-induced HUVEC injury.

The physiological functioning of the endoplasmic reticulum is important for most cellular activities and survival. In response to certain stimuli, endoplasmic reticulum stress is activated in pathological vascular remodeling in cardiovascular diseases (37, 38). The excessive or prolonged stress may result in cell apoptosis and injuries (39). The enhanced expression of endoplasmic reticulum-bounded chaperone protein glucose-related protein (GRP78) is induced in response to endoplasmic reticulum dysfunction. Activating transcription factor-4 (ATF-4) is involved in the upstream signaling pathways in the process of endoplasmic reticulum stress. C/EBP homologous protein (CHOP) can initiate apoptotic events in the setting of severe or prolonged endoplasmic reticulum stress (38). Real-time PCR assay (Figures 4B–D) showed that TCP pretreatments could significantly downregulate the mRNA levels of GRP78, CHOP, and ATF4. Flow cytometry assay (Figure 5) demonstrated that TCP pretreatments could significantly decrease apoptosis proportion compared with the H_2_O_2_ group. Therefore, antioxidant properties of TCPs against H_2_O_2_-induced HUVEC injury resulted from the downregulation of mRNA levels of GRP78, CHOP, and ATF4 and significant decrease of apoptosis proportion. Previous work demonstrated that higher levels of oxidative stress contributed to the activation of endoplasmic reticulum stress, which mediated oxidative stress-induced apoptosis via an extrinsic pathway (40). Therefore, the TCP pretreatment might decrease the oxidative stress in HUVECs, which contributed to the deactivation of endoplasmic reticulum stress and further decreased oxidative stress-induced apoptosis.

## Conclusion

This study analyzed the chemical localization, structural changes, and antioxidant properties of TCPs in tuna head soup. The chemical localization of TCPs was shown by co-localization technique of LSCM. The size distribution and structural changes of TCPs in the boiling process were analyzed by Malvern size analyzer and TEM. These results showed the chemical localization and core–shell structures of TCPs. Moreover, the sizes of TCPs increased with the increase of boiling time. Further, the antioxidant properties of TCPs against H_2_O_2_-induced HUVEC injury were studied by cell viability assay, real-time PCR assay, and flow cytometry assay. TCPs had excellent antioxidant properties against H_2_O_2_-induced cell injury. TCP pretreatments could decrease mRNA levels of GRP78, CHOP, and ATF4 and decrease cell apoptosis proportion and, therefore, increase cell viability. This work provided useful basic information to understanding the colloidal system in foods. In addition, the excellent antioxidant properties of tuna head soup could also promote the potential high-value-added utilization of aquatic by-products.

## Data Availability Statement

The original contributions presented in the study are included in the article/supplementary material, further inquiries can be directed to the corresponding authors.

## Author Contributions

CM: methodology, investigation, and writing—original draft preparation. PL: investigation. NT: conceptualization, project administration, formal analysis, resources, and funding acquisition. XW: conceptualization, project administration, formal analysis, resources, and writing—review and editing. SD: conceptualization and formal analysis. All authors contributed to the article and approved the submitted version.

## Conflict of Interest

The authors declare that the research was conducted in the absence of any commercial or financial relationships that could be construed as a potential conflict of interest.
